# Synoptic Diagnostics of Myeloproliferative Neoplasms: Morphology and Molecular Genetics

**DOI:** 10.3390/cancers13143528

**Published:** 2021-07-14

**Authors:** Dominik Nann, Falko Fend

**Affiliations:** 1 Institute of Pathology and Neuropathology, University Hospital Tübingen, 72076 Tübingen, Germany; dominik.nann@med.uni-tuebingen.de; 2 Comprehensive Cancer Center, University Hospital Tübingen, 72076 Tübingen, Germany

**Keywords:** myeloproliferative neoplasms, trephine biopsy, molecular genetics, bone marrow, differential diagnosis, classification

## Abstract

**Simple Summary:**

The diagnosis of myeloproliferative neoplasms requires assessment of a combination of clinical, morphological, immunophenotypic and genetic features, and this integrated, multimodal approach forms the basis for precise classification. Evaluation includes cell counts and morphology in the peripheral blood, bone marrow aspiration and trephine biopsy, and may encompass flow cytometry for specific questions. Diagnosis nowadays is completed by targeted molecular analysis for the detection of recurrent driver and, optionally, disease-modifying mutations. According to the current World Health Organization classification, all myeloproliferative disorders require assessment of molecular features to support the diagnosis or confirm a molecularly defined entity. This requires a structured molecular analysis workflow tailored for a rapid and cost-effective diagnosis. The review focuses on the morphological and molecular features of Ph-negative myeloproliferative neoplasms and their differential diagnoses, addresses open questions of classification, and emphasizes the enduring role of histopathological assessment in the molecular era.

**Abstract:**

The diagnosis of a myeloid neoplasm relies on a combination of clinical, morphological, immunophenotypic and genetic features, and an integrated, multimodality approach is needed for precise classification. The basic diagnostics of myeloid neoplasms still rely on cell counts and morphology of peripheral blood and bone marrow aspirate, flow cytometry, cytogenetics and bone marrow trephine biopsy, but particularly in the setting of Ph− myeloproliferative neoplasms (MPN), the trephine biopsy has a crucial role. Nowadays, molecular studies are of great importance in confirming or refining a diagnosis and providing prognostic information. All myeloid neoplasms of chronic evolution included in this review, nowadays feature the presence or absence of specific genetic markers in their diagnostic criteria according to the current WHO classification, underlining the importance of molecular studies. Crucial differential diagnoses of Ph− MPN are the category of myeloid/lymphoid neoplasms with eosinophilia and gene rearrangement of *PDGFRA*, *PDGFRB* or *FGFR1*, or with *PCM1*-*JAK2*, and myelodysplastic/myeloproliferative neoplasms (MDS/MPN). This review focuses on morphological, immunophenotypical and molecular features of *BCR-ABL1*-negative MPN and their differential diagnoses. Furthermore, areas of difficulties and open questions in their classification are addressed, and the persistent role of morphology in the area of molecular medicine is discussed.

## 1. Introduction

The current World Health Organization (WHO) classification of tumors of hematopoietic and lymphoid tissue from 2017 classifies myeloid neoplasms by a combination of clinical, morphological, immunophenotypic and genetic features. Thus, an integrated, multimodal approach is needed for precise classification. Although the basis of diagnosis of myeloid neoplasms is still the morphology of peripheral blood and bone marrow aspirate, complemented by cytogenetics, flow cytometry and histology on trephine biopsies, increasing knowledge about molecular alterations has led to a paradigm shift in the classification, with molecular features gaining importance and resulting in entities defined in part, or even exclusively, by recurrent genetic alterations. However, especially in the setting of myeloproliferative and myelodysplastic/myeloproliferative neoplasms, the bone marrow trephine biopsy still has a crucial role to provide additional information about cellularity, histotopography of hematopoietic cells and their maturation, bone marrow stroma and bone structure [1]. Morphological assessment is enhanced by immunohistochemical staining, e.g., for assessing blast counts or highlighting atypical micromegakaryocytes difficult to identify by routine stains. The basis of a good investigation is an adequate biopsy of sufficient length (≥1.0 cm evaluable hematopoietic marrow) with good fixation and decalcification, and without significant aspiration or crush artifacts [1,2,3,4]. Decalcification with EDTA provides an advantage over acid decalcification in better preservation of nucleic acids for molecular studies and antigens for immunohistochemistry.

For the morphological review of a bone marrow trephine with a differential diagnosis of myeloproliferative neoplasm (MPN), hematoxylin and eosin (H&E) and Giemsa stains of good quality, a silver stain optionally complemented by a trichrome stain to evaluate fibrosis, an iron stain and, optionally, periodic acid-Schiff (PAS) and naphthol AS-D chloroacetate esterase (CAE) histochemical stains, render an optimal overview of the different components of hematopoiesis and supporting structures [1]. A basic immunohistochemical panel for myeloid analysis should contain CD34 and CD117 for blast screening and detection of mast cells, CD61 or CD42b for megakaryopoiesis, and CD71 or analogous markers for erythropoiesis, complemented by CD3 and CD20 or equivalent markers for lymphoid cells. Further immunohistochemical markers can be added for specific questions, e.g., CD14 and/or lysozyme for monocytic and monoblastic differentiation [1]. In contrast to other myeloid neoplasms, especially AML and MDS, flow cytometry has less impact on diagnosis and classification of MPN in comparison to morphological and molecular evaluation. Nevertheless, flow cytometry can demonstrate aberrant expression of surface markers, may show evidence of progression, and can help to separate MPN from other chronic myeloid disorders [5,6].

This review focuses on the histology and immunophenotype of *BCR-ABL1*-negative myeloproliferative neoplasms on bone marrow biopsies, including their molecular features and discusses questions, which remain open with regard to the current classification.

## 2. *BCR-ABL1-*Negative Myeloproliferative Neoplasms

The current update to the 4th Edition of the WHO classification of MPN mentions seven entities, including chronic myeloid leukemia-*BCR-ABL1*-positive, chronic neutrophilic leukemia (CNL), polycythemia vera (PV), primary myelofibrosis (PMF), essential thrombocythemia (ET), chronic eosinophilic leukemia (CEL)-not otherwise specified (NOS), and myeloproliferative neoplasm–unclassifiable (MPN-U) [1].

MPNs are clonal hematopoietic stem cell disorders characterized by an increased proliferation of one or more myeloid lineages predominantly arising in adults in their fifties to seventies, but also occasionally occurring in younger age groups including children [1,7,8,9]. In the peripheral blood, the levels of granulocytes, red blood cells, and/or platelets are often elevated due to effective maturation lacking dysplasia, and frequently a hypercellular bone marrow is found. Clinically, a splenomegaly and a hepatomegaly due to extramedullary hematopoiesis are common [1]. MPNs in the chronic phase are unique among myeloid neoplasms in that clinical symptoms and complications are primarily due to the overproduction of mature and functional hematopoietic elements, e.g., causing circulatory problems or thrombotic events. All MPNs, though at very different frequencies, can progress to blast crisis, characterized by >20% blasts in the blood or bone marrow, and development of hematopoietic insufficiency.

Chronic myeloid leukemia (CML) is a molecularly defined disease and requires the detection of the t(9;22) translocation, resulting in the Philadelphia (Ph) chromosome and the *BCR-ABL1* fusion gene [10,11]. By this criterion, CML is set apart from other MPNs, in which molecular features are important, but not sufficient on their own to define an entity; therefore, this review focuses on *BCR-ABL1*-negative MPN.

### 2.1. Chronic Neutrophilic Leukemia (CNL)

This rare MPN shows significant neutrophilia in the peripheral blood and is characterized by hypercellularity of the bone marrow and hepatosplenomegaly. By definition, CNL lacks a *BCR-ABL1* fusion gene and reactive neutrophilia must be excluded [12]. Its true incidence meeting the current diagnostic criteria is unknown. Over 200 cases are published, but if the criteria are applied strictly, some of these cases likely would be excluded [12,13,14]. CNL occurs almost always in older adults with a median of 66 years and a slight male predominance [12,13,14,15]. By WHO definition the blood white blood cell count in peripheral blood is ≥25 × 10^9^/L and segmented neutrophils plus banded neutrophils are ≥80% of the leukocytes [12].

In bone marrow biopsies, the cellularity is typically hypercellular with a dominance of neutrophilic proliferation and a myeloid-to-erythroid ratio of up to 20:1, but without any significant increase of neutrophilic precursors. Both erythropoiesis and megakaryopoiesis show a normal maturation without significant cytological dysplasia [12,13]. If significant dysplasia is found, a diagnosis of atypical CML (aCML) should be entertained [12]. Typical CNL shows no or only minimal fibrosis on reticulin staining [12].

An important differential diagnosis of CNL is a neutrophilic leukemoid reaction in the setting of a plasma cell neoplasia. The WHO classification recommends in patients with leukocytosis and multiple myeloma or monoclonal gammopathy of undetermined significance the demonstration of cytogenetic or molecular clonality in the neutrophil lineage before an additional diagnosis of CNL is made [12,13].

The molecular landscape of CNL is dominated by *CSF3R* mutations, which are found in the majority of CNL cases (64% to 100%) (Table 1) [16,17,18,19,20,21,22], first described in 2013 [23]. *CSF3R* mutations can be divided into different groups: the common membrane proximal mutations, primarily T618I and T615A, resulting in ligand-independent receptor activation, and truncating mutations in the cytoplasmic tail between amino acid 651 and 836, which almost always are compound mutations found in about 25% of patients with membrane proximal mutations, suggesting that the latter on their own lack sufficient oncogenicity [23,24,25]. Patients with T618I mutation show adverse clinical and laboratory features as well as lower overall survival [17]. In addition to *CSF3R* alterations, other genes are mutated, including *ASXL1* (47% to 77%), *SETBP1* (0% to 75%), *SRSF2* (44%), *TET2* (20.5% to 50%), *CALR* (5% to 12.5%), and *JAK2* (8%) (Table 1) [16,17,18,19,20,21,22]. Some of these mutations, e.g., in epigenetic modifier and spliceosome genes, are classic CHIP (clonal hematopoiesis of indeterminate potential) mutations (*DNMT3A, ASXL1, TET2, JAK2, PPMID1, SF3B1, SRSF2, TP53, CBL*, and others). CHIP can be detected in about 10% of all healthy individuals over 65 years of age and is defined as the occurrence of a clonal mutation in at least 4% of hematopoietic cells without evidence of a hematological neoplasm. CHIP is an optional preneoplasia, and is correlated with the development of hematological neoplasms with a probability of 0.5 to 1% per year, especially myelodysplastic syndrome (MDS) and acute myeloid leukemia (AML), depending on the clone size, number of mutations and affected genes [26,27]. Based on the common occurrence of CHIP mutations in CNL, it has been suggested that both CNL and atypical chronic myeloid leukemia (aCML), discussed below, should be considered disorders of clonal hematopoiesis [16].

As mentioned above, a main differential diagnosis of CNL is aCML. There are morphological and laboratory criteria separating the two disorders, including neutrophil precursors ≥ 10% of the leukocytes and the presence of dysgranulopoiesis, which may include abnormal chromatin clumping, as well as dysplasia in other cell lines in aCML. Although in the first study *CSF3R* mutations were observed in 44% of aCML [23], further studies revealed a much lower frequency between 0 and 11%, [19,46,47,48]. There are also other entities which can contain a *CSF3R* alteration. In adult and pediatric acute myeloid leukemia about 0.5 to 1% and about 2 % harbor a *CSF3R* mutation, respectively [23,49,50,51], and in chronic myelomonocytic leukemia (CMML) about 4% show such a mutation [52]. The mutational landscape of aCML is dominated by *ASXL1* mutations (81% to 92%), followed by *SRSF2* (37% to 48%), *TET2* (37%) and *EZH2* (30% to 32%) [16,46]. *SETBP1* mutations have a frequency between 7% and 38% in aCML [16,46,53,54]. In CNL, *SETBP1* is mostly associated with the *CSF3R* mutation, so that despite a high frequency in both entities, the sole occurrence of *SETBP1* is more in line with aCML [13,55]. Due to the overlapping mutational and expression profile of CNL and aCML, and to a lesser extent chronic myelomonocytic leukemia (CMML) and MDS/MPN unclassifiable, and their association with CHIP, it has recently been suggested that these entities might represent a continuum of closely related hematological disorders rather than discrete entities [16].

### 2.2. Polycythemia Vera (PV)

This MPN is characterized by an increase of red blood cell volume and absence of physiological regulation of erythropoiesis as evidenced by low erythropoietin levels. According to current WHO criteria, PV shows elevated hemoglobin concentration (>16.5 g/dL in men, >16.0 g/dL in women), or elevated hematocrit (>49% in men, >48% in women), or increased red blood cell mass (>25% above mean normal predicted value) (Table 2) [56]. It is also a disease of elderly people with a median age of 60 years, and with a slight male predominance with a male-to-female ratio of 1–2:1 [28,56,57,58]. PV can be divided into two phases: the polycythemic phase and the post-polycythemic myelofibrosis phase. The second phase is characterized by cytopenia (including anemia), bone marrow fibrosis, extramedullary hematopoiesis, and hypersplenism [56]. In the initial phase, some patients show obvious thrombocytosis and no elevated hemoglobin/ hematocrit, mimicking ET and designated the pre-polycythemic phase, but subsequent development of raised red blood cell counts and typical bone marrow pathology unmask the PV diagnosis [56,59,60,61].

**Figure 1:**
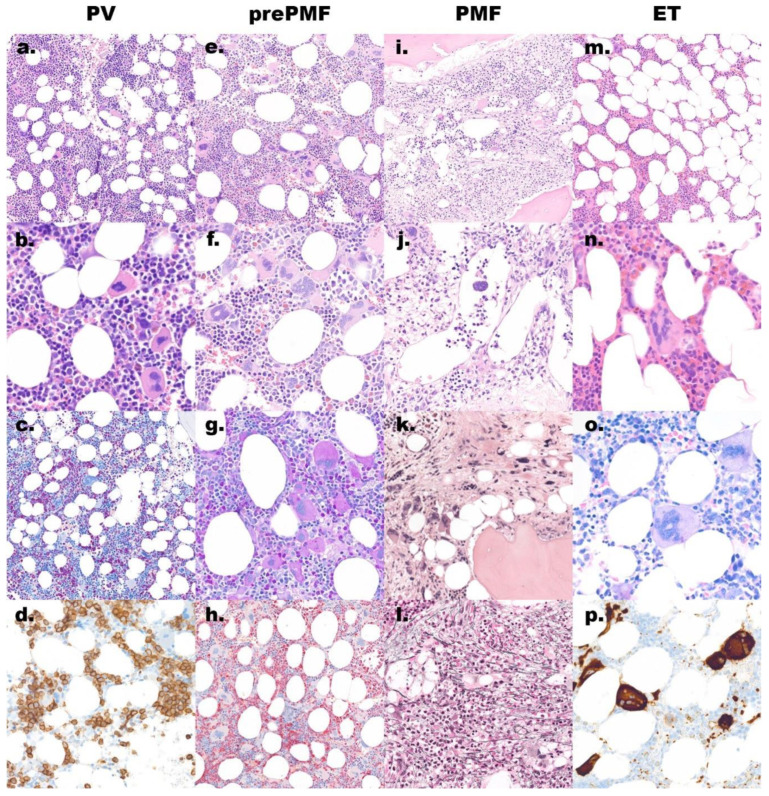
Morphology overview of the main discussed entities. (**a**–**d**) Polycythemia vera (PV) shows a hypercellular bone marrow biopsy: (**a**) hematoxylin and eosin (H&E) stain, original magnification 200×; with increased, morphologically quite variable megakaryocytes: (**b**) H&E stain, original magnification 600×; increased erythropoiesis and reduced granulopoiesis: (**c**) naphthol AS-D chloracetate esterase stain, original magnification 200×; CD71 marks the increased and left shifted erythropoiesis: (**d**) immunoperoxidase, original magnification 400×; (**e**–**h**) Prefibrotic/early primary myelofibrosis (pre-PMF) shows a hypercellular bone marrow: (**e**) H&E stain, original magnification 200×; with increased megakaryocytes with tight clusters and large, atypical, often cloudy nuclei: (**f,g**) H&E and PAS stain, original magnification 400×; the granulopoiesis is often increased: (**h**) naphthol AS-D chloracetate esterase stain, original magnification 200×; (**i–l**) Overt primary myelofibrosis (PMF) shows a hypercellular bone marrow with typical paratrabecular fat tissue: (**i**) H&E stain, original magnification 200×; open sinus with hematopoiesis: (**j**) H&E stain, original magnification 400×; in late stage a reduced cellularity with irregular megakaryocytes lying in a streaming pattern with paratrabecular fat tissue: (**k**) H&E stain; original magnification 400× and increased reticulin fibers: (**l**) silver stain, original magnification 400×; (**m**–**p**) Essential thrombocythemia (ET) has usually a normocellular bone marrow: (**m**) H&E stain, original magnification 200×; with increased megakaryocytes with hypersegmented nuclei: (**n**) H&E stain and (**o**) Giemsa stain, original magnification 600×; the CD61 stain (**p**) marks also these large megakaryocytes, immunoperoxidase, original magnification 400×.

The typical bone marrow biopsy (Figure 1a–d) in the polycythemic phase is hypercellular with a generalized panmyelosis, including the subcortical, normally hypocellular, spaces [56,65,66,67]. Most prominent are the usually normoblastic erythroid precursors, which lie in enlarged islands, and the megakaryocytes, which often show hypersegmented or pleomorphic nuclei and significant size variability, and may form loose, but not tight clusters. Granulopoiesis is in general morphologically unremarkable but increased for age [65,66,68,69]. As mentioned above, the so-called masked/ prodromal PV can mimic ET at the beginning, especially due to increased megakaryopoiesis with often hypersegmented nuclei in the context of high platelet counts and no elevated hemoglobin/hematocrit [60,61,70]. PV has no significant increase in reticulin fiber network in up to 80% of cases, with the remainder showing mild to moderate fibrosis at initial diagnosis, a sign for early progression to post-polycythemic myelofibrosis [65,66,67,71,72,73,74,75]. In nearly all cases of PV no stainable iron can be found in bone marrow aspirate and biopsy. Reactive lymphoid aggregates exist in about one-fifth of cases [66,68,69,75,76]. Post-polycythemic myelofibrosis is characterized by an overt reticulin and collagen fibrosis of the bone marrow with increasing hypocellularity, decreased erythropoiesis resulting in anemia, more clustered megakaryocytes with often hyperchromatic and dysmorphic nuclei, and in the late stage decreased granulopoiesis. Post-PV myelofibrosis occurs in 4.9–6% of cases at 10 years and 6–14% of cases at 15 years [77]. Whether post-polycythemic myelofibrosis can be discerned from PMF remains controversial, but increased cellularity, less prominent atypia and less clustering of megakaryocytes have been identified as features of fibrotic progression of PV [66,68,75,78,79,80]. In addition to anemia, additional clinical criteria are increasing splenomegaly, leukoerythroblastosis, weight loss, night sweats and unexplained fever [78]. Neutrophilic leukocytosis (CNL-like) in this stage is linked to an adverse course of the disease (see below) [81].

Nearly all patients with PV have a *JAK2* (Janus kinase 2) mutation, of which 96% harbor the classic *JAK2* V617F mutation in exon 14 and about 3% an exon 12 mutation (Table 1) [28,29,30,31,32,33]. These lead to constitutive activation of the JAK-STAT pathway and independent growth [82,83]. Both mutations are prognostically similar, but patients with an exon 12 mutation usually show prominent erythroid hematopoiesis and lower leukocytes and platelet counts, whereas a younger age at diagnosis and higher hemoglobin levels have been found in some, but not all studies of these patients [84,85]. In 2014, two cases of *JAK2* V617F and *JAK2* exon 12-negative PVs were described with a *calreticulin* (*CALR*) mutation (52 base pair deletion) in peripheral granulocytes, providing an alternative pathogenesis for *JAK2*-negative PV [64]. Recently, we observed a similar case with a predominance of erythropoiesis, enlarged and irregular megakaryocytes without clustering and a confirmed *CALR* mutation (type 1), which was classified in synopsis with the peripheral blood values (hemoglobin 17.5 g/dL, thrombocytes 565 × 10^9^/L) as a *JAK2^WT^/CALR^mut^* PV.

Patients with PV tend to have higher allelic burden of the *JAK2* mutation in comparison to patients with ET, with about 25 to 30% of PV and 2 to 4% of ET harboring a homozygous *JAK2* mutation, usually due to uniparental disomy. Homozygosity is associated with a more symptomatic course and a higher progression to secondary myelofibrosis in both PV and ET, with a higher risk of thrombotic events in patients with ET [86]. Additionally, an allelic burden ≥ 75% in PV at the time of diagnosis was associated with high-risk disease [87]. In recent years, additional mutations to the driver mutation were detected, especially classic CHIP mutations. In PV, these include *TET2* (10–20%), *ASXL1* (up to 10%), *DNMT3A* (5%), and *SF3B1* (5%), which affect DNA methylation, histone modification and mRNA splicing (Table 1) [34,35,36]. An interesting feature is the time point of acquisition of these secondary mutations and their correlation with the clinical phenotype. When *JAK2* is the first mutation, the patient presents more commonly with *PV*, whereas when *TET2* or *DNMT3A* occur prior to *JAK2*, the phenotype is more likely ET [35,88]. The additional mutations also have a prognostic impact, with *ASXL1*, *SRSF2*, and *IDH2* mutated cases showing an adverse prognosis in PV [36].

### 2.3. Primary Myelofibrosis (PMF)

PMF is a myeloproliferative neoplasm with a dominance of the granulocytic lineage and atypical, large megakaryocytes in the bone marrow, progressive fibrosis and development of significant extramedullary hematopoiesis during the course of the disease. There are two stages of development: initially a prefibrotic/ early stage, followed by an overt fibrotic stage. It is mostly a diagnosis of the sixth and seventh decade of life and the gender distribution is roughly even [62]. About 15% of PV patients, and a small minority of patients with ET, show progression to a myelofibrotic phase, usually many years after primary diagnosis, called secondary myelofibrosis [78,89]. Whereas the prefibrotic stage of PMF frequently shows leuko- and thrombocytosis in the peripheral blood very similar to ET, the typical features of the overt fibrotic stage are increasing anemia, a blood smear with leukoerythroblastosis and teardrop-shaped red blood cells [62].

The molecular landscape of PMF shows one of the typical and disease-defining mutations in JAK2 (virtually always V617F), CALR encoding for calreticulin and MPL (myeloproliferative leukemia virus oncogene) encoding the thrombopoietin receptor. More than half of the cases (about 50–65%) show the JAK2 mutation in exon 14, followed by 25 to 30% with a CALR mutation and 8 to 10% with a MPL mutation (Table 1) [29,37,38]. Calreticulin is a molecular chaperone residing in the endoplasmatic reticulum, and a crucial protein in calcium homeostasis and protein folding [90]. About 80% of all CALR alterations are made up of two main mutations, both affecting exon 9: the first one (type 1) is a long deletion (c.1092_1143del, p.L367fs*46), and the second (type 2) is a short insertion (c.1154_1155insTTGTC, p.K385fs*47). The remaining mutations are grouped with one of the two main alterations based on their similarity [91,92]. In PMF, the type 1 alteration is more common than type 2 (70%/13%), whereas in ET the two types are more equally distributed (51%/39%) [93]. All CALR mutations result in a + 1 frameshift resulting in a new C-terminus lacking the KDEL motif required for retention in the endoplasmatic reticulum. Functional studies have shown that mutant CALR binds to MPL resulting in activation of MPL and thereby a downstream activation of the JAK-STAT pathway [94,95,96,97]. The constitutive MPL activation common to both MPL and CALR mutated MPN, induces megakaryopoiesis and platelet production and explains the thrombocytosis as a common laboratory feature. Patients with a type 1 CALR mutation have a significantly longer survival in comparison to JAK2, MPL, type 2 CALR and the rare triple-negative cases. The absence of a type 1 CALR alteration, or the presence of an additional ASXL1/SRSF2 mutation, were separately prognostic for lower survival. Moreover, patients with the type 1 CALR mutation and additional ASXL1/SRSF2 alterations have a significantly longer survival in comparison to patients with these additional mutations and a nontype 1 alteration, suggesting that type 1 mutations ameliorate the actual negative effect of these additional mutations [98]. The most common *MPL* mutations are point mutations in exon 10 at position 515 (mostly W515L and W515K) within a transmembrane domain of the protein resulting in a gain of function, but other alterations are also described like the S505N mutation [99,100,101,102]. As mentioned above, there are additional secondary mutations in PMF cases, including CHIP mutations such as *ASXL1* (up to 35%), TET2 (20%), *SRSF2* (up to 20%), *U2AF1* (16%), *ZRSR2* (10%), *SF3B1* (10%), *DNMT3A* (5–15%), among others, which are less common (Table 1) [39,40,41,42]. These secondary mutations mainly affect genes involved in DNA methylation, mRNA splicing and histone modification and transcription factors. These secondary mutations, more typically found in CHIP and MDS, are more common in PMF than ET and PV, and might, in part, explain the worse prognosis of PMF. A small number of cases, up to 12% of PMF are so-called triple negative cases lacking the classic driver mutations [34,41]. Deep sequencing identified mutations outside the classic regions of *MPL* exon 10 in about 10% of these triple-negative cases [103]. Although PMF shows the worst prognosis of the three classical Ph− MPN, there is a wide range of clinical behavior, with some patients, especially those with pre-PMF, show a relatively stable course over many years, whereas others progress rapidly and die within a few years after diagnosis [37,89]. In order to stratify patients according to their risk for disease progression and selection of appropriate therapy, including allogeneic stem cell transplant, a variety of risk scores such as the DPISS, MIPSS70 and GIPSS, containing clinical, genetic or a combination of these parameters, have been developed over the years [37].

#### 2.3.1. Prefibrotic/Early Primary Myelofibrosis

The diagnosis of a prefibrotic PMF is based on a set of clinical, laboratory, morphological and molecular criteria (Table 2) [62]. Clinically, these include anaemia (without any other cause), thrombocytosis, leukocytosis above 11 × 19^9^ /L, splenomegaly or an increase of lactate dehydrogenase (LDH) [62,104]. Thrombocytosis can be quite high, and if the remaining blood values are normal or borderline the differentiation from ET is difficult and a bone marrow biopsy is mandatory [105,106,107]. About 30% to 50% of cases with PMF are diagnosed in this prefibrotic stage [73,108,109,110].

The bone marrow is hypercellular and shows a proliferation of megakaryopoiesis and granulopoiesis with usually decreased erythropoiesis (Figure 1e–h). Granulopoiesis can be left shifted, but myeloblasts are usually not increased [109,110,111]. The most prominent and distinctive feature of prefibrotic PMF is megakayopoiesis. Distribution and morphology are important criteria for the distinction from ET. The megakaryocytes often form dense clusters and are commonly found next to vessels and bone trabeculae. Megakaryocytes are usually enlarged but scattered small cells may also be present; they show an increased nuclear/cytoplasmic ratio, an abnormal density of the chromatin with cloud-like nuclei and in part also dispersed naked nuclei. In contrast to the other MPN, megakaryocytes are distinctly more atypical in pre-PMF [69,70,73,76,107,110,112]. For diagnosis of this stage, a maximum of grade 1 fibrosis may be present according to the semiquantitative bone marrow fibrosis grading system [67,113]. In up to 20% of BM, trephines reactive lymphoid aggregates can be found [107,110].

#### 2.3.2. Overt Primary Myelofibrosis

More than 50% of patients with PMF are diagnosed in the overt fibrosis stage [73,109,110,114]. The main feature of this stage is the clear increase in reticulin or collagen fibers (grade 2 or 3), frequently associated with osteosclerosis (Table 2) [67,113]. The bone marrow itself (Figure 1i–k) is usually normo or hypocellular and only rarely hypercellular, with reduced hematopoiesis and areas of loose connective tissue. A characteristic feature of PMF is the redistribution of fat cells along the bone trabecules. Megakaryocytes are very conspicuous with numerous atypical shapes and frequent clustering. Granulopoiesis, and especially erythropoiesis, are often significantly reduced. Important stromal changes are a high density of vessels and dilated sinuses with characteristic intrasinusoidal hematopoiesis [110,115,116,117]. Late stage PMF cases may show almost complete absence of hematopoiesis with extensive fibrosis, including collagen and osteosclerosis with irregular apposition of osteoid. As the disease progresses, the occurrence of extramedullary hematopoiesis becomes more evident, mainly in the spleen and less frequently in the liver and other organs. In the spleen, there is hyperplasia of the red pulp, with cells of maturing trilineage hematopoiesis. The appearance of dense monomorphic round cell areas and nodules should suggest a differential diagnosis of transformation to an extramedullary manifestation of AML/myelosarcoma and trigger additional immunohistochemical staining for blasts (CD34, CD117) [118,119,120,121,122].

### 2.4. Essential Thrombocythemia (ET)

ET is characterized by dominance of megakaryopoiesis with prominent thrombocytosis in the peripheral blood [63]. The WHO classification requires a platelet count ≥ 450 × 10^9^ /L, a typical bone marrow morphology, the exclusion of other WHO-defined diseases such as CML, PV and PMF or other myeloid neoplasms, and one of the driver mutations in *JAK2*, *CALR*, or *MPL*. In their absence, the WHO classification calls for the presence of another clonal marker or the exclusion of reactive thrombocytosis (Table 2) [63]. Like the other classical MPNs, ET is also a disease of older people, with a peak between 50 and 60 years and a minimal predilection of women; especially in women there is a further peak around 30 years [38,105,123,124,125]. ET can also be observed in children. Like in PV, there is a small risk to develop a so-called post-ET myelofibrosis, ranging from <0.8–4.9% after 10 years [43,77]. The varying frequencies of post-ET myelofibrosis in part can be explained in the difficult separation of ET from pre-PMF.

A bone marrow biopsy (Figure 1m–p) is, in general, normocellular, and only few cases are hypercellular [61,67,76]. The most important and prominent finding is the increased megakaryopoiesis with many big, “aged” megakaryocytes, showing large cytoplasm and lobated and hypersegmented “staghorn” nuclei, which are mostly distributed singly throughout the bone marrow, sometimes in loose clusters. The erythropoiesis and granulopoiesis, in general, are normal, but due to hemorrhage, erythropoiesis may be left shifted [63,105,106,126,127,128]. By definition, significant fibrosis is absent, and only grade 1 fibrosis is allowed, which occurs in less than 5% of cases. [38,67,74,76,106,123,126,127,129].

The differential diagnoses are broad and the morphological findings in the bone marrow trephine are crucial. With increase in granulocytic and erythroid lineage and the *JAK2* V617F mutation, a masked/prodromal PV should be considered [60,61,70]. In comparison to PMF, megakaryocyte clusters are rare and normally loose, and the megakaryocytes are not atypical, without clumped chromatin and cloud-like nuclei [107,112,126,127,130]. In the presence of anemia, an MDS/MPN with ring sideroblasts and thrombocytosis (RARS-T) needs to be considered.

A diagnosis of post-ET myelofibrosis requires a documented diagnosis of ET and a bone marrow fibrosis of grade 2 or 3, and additionally at least two more criteria of the following are needed: anaemia, leukoerythroblastosis, increasing splenomegaly, elevated LDH value, or two or three of the following symptoms: >10% weight loss within six months, night sweats or unexplained fever above 37.5°C [63,78].

The mutational landscape of ET is similar to PMF with regard to driver mutations: about 50–60% of cases harbor the *JAK2* mutation V617F, but no *JAK2* exon 12 mutations, up to 30% show *CALR* and 3–8% *MPL* mutations; up to 15% are “triple-negative” for the classic MPN driver mutations and are difficult to separate from reactive thrombocytosis (Table 1) [29,34,38,43,44]. As mentioned above, cases which acquire *TET2* or *DNMT3A* mutations before the *JAK2* mutation more often have an ET phenotype [35,88], and the two different types of *CALR* mutations are more balanced in ET vs. PMF [93]. The type 1 *CALR* mutation shows a significantly higher risk of transformation to myelofibrosis in ET, and patients with both *CALR* types show a higher platelet count, lower hemoglobin and leukocyte values in comparison to *JAK2*-positive ET [131,132]. Patients with *CALR* mutated ET are also younger, have a lower risk for thrombosis, and show no progression to PV (vs. 29% at 15 years), assuming that *JAK2*-positive ET and PV are different stages/phenotypes of a single disease, and *CALR*-positive ET is a different nosological entity [133]. Atypical *JAK2* mutations are found in single, classic triple-negative ET cases including V625F and F556V [103]. Although at a lower frequency than in PMF, additional mutations including CHIP mutations, occur in ET, including *TET2* (10–15%), *ASXL1* (5–10%), *DNMT3A* (5%), *SF3B1* (3%), and others with lower frequencies (Table 1) [34,36]. Some additional mutations show an adverse prognostic impact in ET, including *SH2B3*/*LNK*, *SF3B1*, *U2AF1*, *TP53*, *IDH2*, and *EZH2* [36].

### 2.5. Chronic Eosinophilic Leukemia, Not Otherwise Specified (CEL, NOS)

CEL, NOS is still a diagnosis of exclusion and is characterized by an increase of eosinophils and their precursors in the bone marrow, the peripheral blood and tissue, and consecutive damage of organs [134]. The diagnostic criteria for CEL in the WHO classification are an eosinophil count ≥ 1.5 × 10^9^ /L and evidence of a clonal cytogenetic/molecular genetic abnormality, or blast cell count of ≥2% of cells in the peripheral blood or ≥5% in the bone marrow. Additionally, other WHO-defined neoplasms, including all other MPNs, CMML, aCML, and especially myeloid/lymphoid neoplasms with eosinophilia and gene rearrangement of *PDGFRA*, *PDGFRB* or *FGFR1,* need to be excluded, and no *PCM1*-*JAK2*, *ETV6*-*JAK2* or *BCR*-*JAK2* fusion, blasts ≥ 20% or inv(16)(p13.1;q22), t(16;16)(p13.1;q22), or t(8;21)(q22;q22.1) are allowed for a diagnosis of CEL [134]. If eosinophilia persists for ≥6 months, and any other MN and reactive eosinophilia have been excluded, a diagnosis of idiopathic hypereosinophilic syndrome (HES) is made in the absence of both an increase in blasts and clonal cytogenetic/molecular genetic abnormality [134,135,136]. Without any evidence of organ damage or dysfunction relatable to tissue hyereosinophilia, the term “(idiopathic) hyereosinophilia” is suitable [134,135,136]. Due to the diagnostic difficulties in differentiating CEL, NOS from other related eosinophilic diseases, valid epidemiological data are rare, but the disease seems to be more common in men and occurs in median in the seventh decade [45,137].

The bone marrow biopsy in CEL NOS is hypercellular with a dominance of eosinophils with mostly normal maturation. The amount of myeloblasts can be increased above 5%, another defining criterion for CEL in the absence of cytogenetic or molecular genetic alterations. The red cell lineage and the megakaryocytes are typically normal, but some dysplastic changes can occur. Charcot-Leyden crystals can be present. Only rare cases show relevant fibrosis (grade 2/3) [45,134,137,138].

For a diagnosis of CEL, NOS extensive molecular analyses are needed, including mutational analysis, RNA-based fusion examination or fluorescence in situ hybridization (FISH) for rearrangement analysis, and cytogenetic studies. Typical nonspecific cytogenetic alterations in CEL, NOS are trisomy 8, loss of chromosome 7, or isochromosome 17 [134,139,140]. Next-generation sequencing identified recurrent mutations in primary idiopathic HES, which showed no significant difference in disease-specific survival to CEL, NOS and, therefore, could be reclassified as CEL, NOS. The identified mutations were *ASXL1* (43%), *TET2* (36%), *EZH2* (29%), *SETBP1* (22%), *CBL* (14%), and *NOTCH1* (14%) [45]. A further study showed known or predicted pathogenic mutations in *TET2*, *ASXL1*, *KIT*, *IDH2*, *JAK2*, *SF3B1* and *TP53* [141]. In 2018, *STAT5B* N642H was identified as an additional hotspot mutation in the setting of eosinophilia. The study also showed additional mutations in most cases; patients with a solitary *STAT5B* or with a further *SF3B1* mutation had a significantly better overall survival in comparison to patients with other additional alterations [142]. Since in elderly persons, many of these mutations with the exception of *STAT5B* can occur as CHIP mutations, it is recommended to eliminate all possible reasons of reactive eosinophilia before making a CEL, NOS diagnosis based on these alterations in an older person [134]. Currently, cases with rearrangements of *ABL1*, *FLT3* and *JAK2*, with the exception of *PCM1-JAK2,* are also classified as CEL, NOS even though they show similarities and often indistinguishable clinical features to the category of “myeloid/lymphoid neoplasms with eosinophilia and fusion genes”, indicating that these cases might be classified differently in the future [135,143]. One additional interesting alteration is the *ETV6*-*ABL1* fusion, which can occur both in children predominantly as acute lymphoblastic leukemia (ALL), and in adults mostly as myeloid neoplasm (MPN-U or AML) strongly reminiscent of *BCR*-*ABL1* positive CML [135,144]. All MPN and AML cases showed eosinophilia, but only a minority of the ALL cases. Therefore, all cases of *BCR-ABL1*-negative MPN resembling CML should additionally be analyzed for this *ETV6*-*ABL1* fusion [144]. Of note, the formation of this fusion gene needs a minimum of three chromosomal breaks, thus routine karyotyping is frequently noncontributory and detailed molecular analysis is needed [144,145]. In conclusion, the detection of one of the disease-specific genetic alterations described above leads to a specific diagnosis associated with eosinophilia instead of CEL, NOS.

### 2.6. Myloproliferative Neoplasm, Unclassifiable (MPN-U)

This category is a waste basket category of cases, which show clear features (clinical, laboratory, morphological, molecular) of MPN, but cannot be categorized to any specific WHO-defined entity, or show overlap between entities. Any disease-defining genetic alterations exclude the diagnosis of MPN-U, such as *BCR*-*ABL1* and *PCM1*-*JAK2*, and rearrangements of *PDGFRA*, *PDGFRB*, or *FGFR1*. [146]. Larger series diagnosed 10–15% of all MPNs as unclassifiable [147,148,149], but accurate analysis including clinical, morphological and molecular characteristics should reduce the frequency to less than 5% [146,147,150,151]. Molecular analysis identifies the same driver mutations as in other MPNs. Gianelli et al. found a *JAK2* mutation in 71.8%, in 11.3% a CALR alteration (type 1: 5.6%, type 2: 2.8%, others: 2.8%), and in 2.8% a *MPL* mutation [147]. As mentioned above, some cases of MPN-U may contain a t(9;12) with *ETV6-ABL1* fusion. Altogether, the category of MPN-U is quite heterogeneous and reflects the biological continuum between the defined entities. Nevertheless, a careful examination is needed to diagnose or exclude a distinct entity before making an MPN-U diagnosis [146,147].

## 3. Special Issues and Still Open Questions

### 3.1. Early Stage Classic Ph− MPN–Clinico-Pathological Versus Molecular Classification

With increasing knowledge about the molecular profiles of classic Ph− MPN, it has become clear that the type of driver mutation, the presence of additional mutations, and their sequence, have a significant influence on prognosis. Furthermore, differentiation between early-stage MPN can be difficult on a clinical morphological basis. For example, a major morphological challenge is the differential diagnosis between essential thrombocythemia and prefibrotic PMF, which show significant differences in progression and survival in many, but not all published studies. The 15-year survival rate is 59% vs. 80% in prefibrotic PMF and ET, respectively; the leukemic transformation rate after 15 years is 11.7% vs. 2.1%, the progression to overt PMF is 16.9% to 9.3%, and the survival of ET patients was similar to the general European population [123]. However, studies from different groups have shown conflicting results concerning reproducibility of the WHO criteria for the separation of these entities and the prognostic impact [152]. Therefore, progression rates for ET and pre-PMF show significant variation in different studies, depending on diagnostic criteria (WHO versus polycythemia vera study group (PVSG)) and morphological evaluation. Therefore, changes in diagnostic criteria, mainly a stronger inclusion of driver mutation status and secondary mutations, have been advocated [37,153]. Given the importance of mutation order for the clinical phenotype of MPN, as mentioned above, examining the order of appearance of secondary mutations based on allelic frequencies might help to predict disease progression better [35,88]. However, despite the increasing weight of molecular markers, clinico-pathological classification remains important, e.g., as demonstrated for the triple negative MPN group. Whereas triple negative ET showed the best overall survival in a univariate, but not in multivariate analysis [154], triple negative PMF showed the worst median overall survival of all PMF [155]. In order to reduce the influence of interobserver variation, advanced image analysis tools have been used to address the morphological separation of Ph− MPN [156].

A related issue showing the importance of clinico-pathological correlation is the occurrence of *JAK2* mutations in individuals without clinical or laboratory evidence of an MPN, which in this setting is considered a CHIP mutation [157]. It is currently unclear how many of these individuals will progress to develop manifest MPN, and what are the underlying mechanisms.

### 3.2. Myeloproliferative Neoplasms with Multiple Driver Mutations

Although the classical driver mutations including *JAK2, CALR, MPL* and *BCR-ABL1* usually are mutually exclusive, rare cases of chronic myeloid neoplasms with multiple driver mutations have been observed. One group of cases shows, in addition to the classic *BCR*-*ABL1* fusion, a *JAK2* or *CALR* mutation. The CML-associated thrombocytosis and leukocytosis often obscure the Ph– MPN, which is only discovered after successful treatment of the CML with tyrosine kinase inhibitors [158,159]. The temporal sequence of these mutations is variable, with the *BCR*-*ABL1* fusion preceding or following a *CALR* or *JAK2* alteration. In the latter case, classic ET or pre-PMF features in the bone marrow are superseded by the classic CML morphology and leukocytosis, and reverse back to the old morphological picture upon successful treatment [160,161,162]. Although the two alterations potentially might reside in the same clone, the evidence of these cases indicates that *BCR-ABL1* and *JAK2* or *CALR* mutations reside in distinct clones. In addition, rare cases with combinations of *JAK2*, *MPL* or *CALR* mutations have been observed [159].

### 3.3. Genetic Overlaps with Other Chronic Myeloid Neoplasms

Similar to the phenomenon described above, MPN may show genetic alterations usually associated with other chronic myeloid neoplasms, and vice versa. Typical MPN driver mutations can occur in the setting of other disorders, such as in myelodysplastic syndromes. [159]. Classification in these unusual cases can be difficult but should rely on the aggregated clinical and pathological features. Examples described in the Workshop Report of the 2017 EAHP/SH meeting included cases of ET with del(5q) or PMF with *SF3B1* p.K666N mutation and ring sideroblasts, in addition to a canonical *JAK2* V617F mutation. Based on morphology, peripheral blood counts and other clinical features, these cases were classified as MPN. On the other hand, cases with classic MDS features may show additional *JAK2* or *MPL* mutations. As long as the clinical and morphological criteria for an MDS are met, classic MPN mutations can occur without the diagnosis being changed, and may represent a background CHIP mutation.

### 3.4. Unusual Types of Progression of MPN

Increasing myelofibrosis, called secondary MF in ET and PV, or transformation to acute leukemia, sometimes in the form of extramedullary myelosarcoma or pure erythroid leukemia, are the two traditionally recognized forms of disease progression in MPN [163].

As briefly mentioned above, neutrophilic leukocytosis can occur in PV patients around the time point of post-polycythemic myelofibrosis [81]. These patients showed persistent absolute leukocytosis of ≥13 × 10^9^ /L (median: 25.1 × 10^9^ /L) with neutrophilic granulocytes usually ≥75% of all white blood cells. The bone marrow presented a prominently increased myeloid to erythroid ratio simulating CNL or CML, but *BCR-ABL1* fusions or additional mutations in *CSF3R*, *SETBP1*, or *SRSF2,* potentially explaining the leukocytosis, could not be detected [78]. In comparison with a cohort without leukocytosis in the postpolycythemic myelofibrosis phase, the first group had a shorter overall survival (median overall survival 181 vs. 252 months).

Another unusual type of progression heralding worse prognosis is the development of absolute monocytosis (>1 × 10^9^ /L) in patients with PMF [164]. Interestingly, bone marrow biopsies revealed a prominent myelomonocytic increase resembling CMML after the onset of monocytosis instead of the characteristic PMF morphology. Of note, cases of PMF with monocytosis at onset may be misdiagnosed as CMML if bone marrow biopsy and testing for MPN driver mutations are not performed [165].

### 3.5. How Much Molecular Testing Is Needed for a Diagnosis of MPN?

Recent technical advances have made high throughput molecular testing, including next generation sequencing, more accessible for many clinical laboratories, and have resulted in its widespread use for the diagnostic workup of MPN. Limited approaches such as single or few gene assays for the main drivers *BCR*-*ABL1*, *JAK2*, *MPL* and *CALR* can still serve as the method of choice in straightforward cases of MPN, but unusual clinico-pathological features, or absence of MPN driver mutations, should prompt more comprehensive testing. In addition, detection of the nonspecific disease-modifying genetic alterations described above may serve for improved prognostication, especially in PMF. Ultimately, more comprehensive genotyping will allow a personalized risk assessment for patients with MPN and better therapeutic stratification. For cases lacking typical driver mutations, translocations involving *PDGFRA/B*, *FGFR1* and *JAK2,* and potentially AML-type alterations, should be investigated in addition to mutations found in myeloid neoplasms (MN) in general. In addition to BM aspirates and peripheral blood leukocytes, EDTA-decalcified bone marrow biopsies are also suitable for extensive testing, including RNA analysis for translocation detection.

Cases requiring more extensive analyses are, for example: MN with clinical/morphological/phenotypical overlaps, such as MPN-U and MDS/MPN-U; MDS with fibrosis or systemic mastocytosis with associated hematological neoplasm; MN presenting as suspected accelerated phase or blast crisis to identify an underlying/antecendent chronic MPN, in the setting of a relapse or progression/transformation to identify the clonal evolution and resistance mutations, and after allogeneic stem cell transplantation to separate a relapse or a second neoplasm from reactive changes.

**Figure 2:**
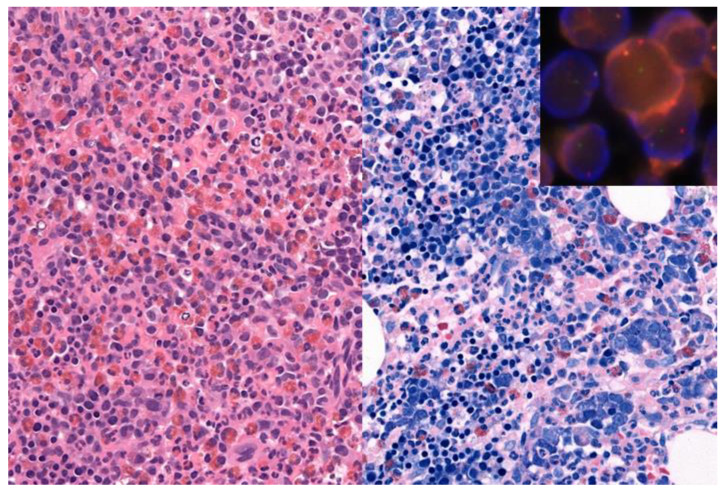
71-year-old man with a hypercellular bone marrow biopsy with eosinophilia (left part: hematoxylin and eosin staining, original magnification 400×) and prominent left shifted erythropoiesis (right part: Giemsa staining, original magnification 400×). Insert: Fluorescence in situ hybridization showed a JAK2 rearrangement with a JAK2 break apart probe. Next generation sequencing identified a PCM1(36)-JAK2(11) rearrangement, in addition to DNMT3A and a NRAS mutations, resulting in a diagnosis of a myeloid/lymphoid neoplasm with PCM1-JAK2 rearrangement.

An example for the value of comprehensive testing is shown in Figure 2. A 71-year-old man showed leukocytosis with left shift, eosinophilia and an elevated LDH of 500, raising a differential diagnosis of MPN versus a myeloid neoplasm with eosinophilia. The bone marrow biopsy was hypercellular with eosinophilia, marked left shifted erythropoiesis, fibrosis grade 1, and mild increase in mast cells, but no blast increase. The initial molecular analyses showed no evidence for *BCL*-*ABL1*, *PDGFRA* or *PDGFRB* alterations or the *JAK2* mutation. Additional extended molecular analysis, including NGS-based search for fusions using RNA from a formalin-fixed, paraffin-embedded bone marrow biopsy, identified a *PCM1(36)-JAK2(11)* rearrangement, subsequently confirmed by FISH and the presence of a t(8;9)(p22;p24) in conventional cytogenetics, resulting in a diagnosis of a myeloid/lymphoid neoplasm with *PCM1-JAK2* rearrangement, a provisional entity newly incorporated into the update of the 4th Edition of the WHO classification.

## 4. Conclusions—The Superior Value of a Synoptic Diagnosis

Myeloid neoplasms are diagnosed by a combination of clinical, morphological, immunophenotypic and genetic features in the current WHO classification, and an integrated, multimodal approach is needed for a precise and clinically applicable diagnosis. Although genetic features will gain further importance, and more MN likely will primarily be genetically defined in the future, clinical features and morphology on the bone marrow trephine biopsy, especially in the setting of MPN and MDS/MPN, still have a crucial role to provide additional information about cellularity, histotopography and distribution of hematopoietic cells, with bone marrow stroma and bone structure remaining important for classification [1]. Molecular features, on the other hand, require integration with clinical and laboratory features, as well as morphology and immunophenotyping, in order to avoid misclassification of MN. Many genetic alterations lack specificity for a certain entity, and background CHIP mutations, or unusual mutational combinations, present diagnostic pitfalls best avoided by a synoptic approach. Novel approaches currently tested in the research setting, such as integrating multiomics and deep learning algorithms for disease classification, will provide new insights for classification and therapy. This review summarizes the salient clinical, morphological and genetic features of Ph− MPN and their differential diagnosis from a practical standpoint, and highlights open questions to be addressed in the future evolution of MPN classification.

## Figures and Tables

**Table 1:** Main genetic alterations in myeloproliferative diseases.

Entity	Main Genetic Alterations	Frequent Additional Genetic Alterations	References
CNL	*CSF3R* (64–100%)	*ASXL1* (47–77%) *SETBP1* (0–75%) *SRSF2* (44%) *TET2* (20.5–50%) *CALR* (5–12.5%) *JAK2* (8%)	[16,17,18,19,20,21,22,23]
PV	*JAK2* V617F (96%) *JAK2* exon 12 (3%)	*TET2* (10–20%) *ASXL1* (up to 10%) *DNMT3A* (5%) *SF3B1* (5%)	[28,29,30,31,32,33,34,35,36]
PMF	*JAK2* V617F (50–65%) *CALR* (25–30%) *MPL* (8–10%)	*ASXL1* (up to 35%) *TET2* (20%) *SRSF2* (up to 20%) *U2AF1* (16%) *ZRSR2* (10%) *SF3B1* (10%) *DNMT3A* (5–15%)	[29,37,38,39,40,41,42]
ET	*JAK2* V617F (50–60%) *CALR* (up to 30%) *MPL* (3–8%)	*TET2* (10–15%) *ASXL1* (5–10%) *DNMT3A* (5%) *SF3B1* (3%)	[29,34,36,38,43,44]
CEL	-	*ASXL1* (43%) *TET2* (36%) *EZH2* (29%) *SETBP1* (22%) *CBL* (14%) *NOTCH1* (14%) *STAT5B* (?)	[45]

CEL: chronic eosinophilic leukemia. CNL: chronic neutrophilic leukemia. ET: essential thrombocythemia. PV: polycythemia vera. PMF: primary myelofibrosis.

**Table 2:** Diagnostic criteria for the main Ph−myeloproliferative neoplasms [56,62,63].

**Polycythemia Vera**	**Prefibrotic Myelofibrosis**	**Overt Myelofibrosis**	**Essential Thrombocythemia**
**Clinical and Laboratory Features**			
Hypertension, thrombotic events **Hb >16.5/16 g/dL (M/F) or Hk >49/48% (M/F)** Low erythropoietin	Splenomegaly Anemia Leukocytosis ≥ 11 × 10^9^ /L Elevated LDH	Splenomegaly Anemia Leukocytosis ≥ 11 × 10^9^/L Elevated LDH Leucoerythroblastosis	**Platelets ≥ 450 × 10^9^/L** **Other MPNs excluded**
**Morphology**			
**Hypercellular marrow with tri-lineage hyperplasia** Pleomorphic MEGs with size variability No stainable iron 80% without fibrosis	**Hypercellular marrow with atypical MEG proliferation (cloud-like nuclei) and dense clusters** Increased GRAN and frequently reduced ERY Fibrosis ≤ 1	**Atypical proliferation of MEG** Variable cellularity (including subtotal aplasia), peritrabecular fat, intrasinusoidal hematopoiesis, osteosclerosis **Fibrosis ≥ 2**	**Mostly normocellular** **Normal ERY and GRAN** **Increased atypical Megs with hypersegmented nuclei** (**staghorn**) **No fibrosis** (**rare G 1**)
**Molecular Features ^§^**			
***JAK2*** V617F or Exon 12 mutation *	***JAK2*** **, *CALR* or *MPL* mutation or other clonal marker**	***JAK2*** **, *CALR* or *MPL* mutation or other clonal marker**	***JAK2*** **, *CALR* or *MPL* mutation or other clonal marker**
**Differential Diagnosis**			
Reactive Polyglobulia	ET RARS-T	Post-PV or post-ET MF MDS-F	RARS-T pre-PMF pre-polycythemic PV

Major criteria according to the WHO classification are in bold. * rare cases of CALR mutated PV have been described [64]. ^§^ see Table 1 for mutation frequencies. MEG: megakaryocytes. GRAN: granulopoiesis. ERY: erythropoiesis. Hb: hemoglobin. Hk: hematocrit. LDH: lactate dehydrogenase. RARS-T: myelodysplastic/myeloproliferative neoplasm with ring sideroblasts and thrombocytosis (MDS/MPN-RS-T). MDS-F: myelodysplastic syndrome with fibrosis.
